# Rapid solvent-evaporation strategy for three-dimensional cobalt-based complex hierarchical architectures as catalysts for water oxidation

**DOI:** 10.1038/s41598-019-51979-z

**Published:** 2019-10-30

**Authors:** Hong Jiang, Hao Zhang, Qiaoling Kang, Haifeng Ma, Yinlin Tong, Feng Gao, Qingyi Lu

**Affiliations:** 1 Department of Materials Science and Engineering, Collaborative Innovation Center of Advanced Microstructures, Nanjing University, Nanjing, 210093 P. R. China; 2 State Key Laboratory of Coordination Chemistry, Coordination Chemistry Institute, Collaborative Innovation Center of Advanced Microstructures, Nanjing National Laboratory of Microstructures, School of Chemistry and Chemical Engineering, Nanjing University, Nanjing, 210093 P. R. China

**Keywords:** Electrocatalysis, Synthesis and processing

## Abstract

It is a challenging task to seek a highly-efficient electrocatalyst for oxygen evolution reaction (OER) of water splitting. Non-noble Co-based nanomaterials are considered as earth-abundant and effective catalysts to lower overpotential and increase polarization current density of OER. In this work, we reported, for the first time, a “rapid solvent-evaporation” strategy for the synthesis of three-dimensional (3D) cobalt complex hierarchical architectures constructed by two-dimensional (2D) nanosheets. The 3D structured cobalt complexes have excellent performances in catalyzing OER with lower onset potential, overpotential, Tafel slope and better stability than commercial IrO_2_. Superior electrochemical performances would be beneficial from the unique 3D structure. This extremely simple method for 3D Co complex with good OER activities makes the complex be promising commercial OER catalyst to replace earth-rare and expensive IrO_2_.

## Introduction

Hydrogen is considered as a promising energy carrier to address issues of global energy security, environmental emissions and sustainability^1–3^. Electrocatalytic water splitting is considered an efficient technology for hydroten production, which consists of hydrogen evolution reaction (HER) on the cathode, and oxygen evolution reaction (OER) on the anode^4^. In the process of OER, large overpotentials are required to promote the reaction, which increases the cost of hydrogen production. So far expensive Ru- and Ir-based compounds, such as Ru, Ir, RuO_2_ and IrO_2_ have reported to efficiently catalyze the OER process^5^. However, high cost and poor durability of these noble catalysts restricts their large-scale application^6^. It is imperative to develop highly efficient and low-cost electrocatalysts to lower the overpotential and accelerate the OER reaction.

Co-based compounds have emerged as a kind of non-noble metal catalysts for OER since Nocera reported that cobalt (II)/phosphate could be an efficient oxygen-evolving catalyst in 2008^7^. Until now, considerable efforts have been devoted to Co-based OER electrocatalysts, including Co oxides^8,9^, Co-based layered metal hydroxides^10–12^, Co phosphides/phosphates^13,14^, Co sulfides/selenides^15,16^, Co oxyhydroxides^17,18^ and cobalt nitrides^19–21^. Previous reports prove that the active sites for OER catalysis are likely from amorphous overlayers comprising cobaltate aggregates instead of the native oxide^22,23^. However, these reported catalysts usually focus on the inorganic cobalt compounds, rare on the cobalt complexes, though the complexes have structural topology and potential functions for gas adsorption, catalytic action and selective adsorption^24^.

Recently, multifunctional three-dimensional (3D) architectures assembled by low-dimensional blocks, such as 0D nanoparticles^25^, 1D nanorods^26^, and 2D nanosheets^27,28^, have drawn wide attention owing to the novel properties produced by synergistic effects of building blocks. Assembling 2D nanosheets into 3D structure not only maintains the intrinsic performances of the 2D nanostructures but also synergistically avoids the accumulation of 2D nanosheets that would dramatically weakens the excellent properties of nanounits^29^. However, according to previous reports, the construction of 2D nanosheets-assembled 3D architectures usually depends on the guide of small molecules or macromolecules, and requires rigorous conditions and techniques, which restrict their wide application as practical methods^30,31^. Therefore, it is highly necessary to develop a facile and efficient strategy for preparing novel 3D structures. In this work, by developing a facile and efficient method called “rapid solvent-evaporation” (RSE) strategy, we synthesized 3D cobalt complex hierarchical architectures assembled by 2D nanosheets. This facile RSE method evaporates solvent quickly during reaction process under high-temperature, resulting in the formation of novel 3D cobalt complex hierarchical architectures, which are completely different from those obtained at slow evaporation processes. More importantly, as a new type Co-based catalyst, the resulted 3D cobalt complexes show excellent catalyzing OER performances in water oxidation compared to commercial IrO_2_.

## Experimental

### Materials

All reagents were of analytical grade and used without further purification. Cobalt acetate and urea were obtained from Sinopharm Chemical Reagent Co. Ltd. Ethanol was purchased from Xilong Scientific Co. Ltd. High purity nitrogen was obtained from Nanjing Shangyuan Industrial Gas Plant. Distilled water was utilized in all experimental procedures.

### Synthesis of the 3D Co complex architectures

Typically, 50 mg of urea was mixed with 1 mL of cobalt acetate aqueous solution (40 mg/mL) in a 4 mL glass bottle. Then, the uncovered bottle was transferred into a drying oven and maintained at 200 °C. The solvent evaporated quickly in 5 min. After the solid was further kept at 200 °C for 1.5 h, blue Co complex (CC-B) was collected at the bottom of the bottle. Final green 3D Co complex (CC-G) was obtained after CC-B was washed alternately with deionized water and ethanol and dried overnight at 80 °C. Schematic diagram of experimental preparation process is presented in Fig. S1.

### Synthesis of SE-100 and SE-150

Comparatively, the bottles with the mixing solution of urea and cobalt acetate were put in drying oven at 100 °C and 150 °C for 1.5 h for slow evaporation. The obtained pink products were washed alternately with deionized water and ethanol, dried overnight at 80 °C and designed as SE-100 and SE-150, respectively.

### Synthesis of CC-air and CC-N_2_

CC-air and CC-N_2_ were prepared through heat annealing treatment of 3D CC-G at the temperature of 600 °C for 3 h in the air or N_2_ atmosphere with a heating rate of 2 °C/min.

### Characterizations

Scanning electron microscopy (SEM) was performed on Hitachi S-4800 at 10 kV. Transmission electron microscopy (TEM) images were obtained by using a JEOL JEM-2100 transmission electron microscope operating at 200 kV. Powder X-ray diffraction (XRD) patterns were collected by using a Bruker D8 ADVANCE diffractometer with CuKα radiation (λ = 1.5418 Å). Infrared spectra were obtained on Fourier transform infrared spectroscopy (FT-IR, Nicolet 6700, Thermo Company). Thermogravimetric analysis of the powders were preformed in air on Pyris Diamond TG/ DTA (Perkin-Elmer). The flow rate of air was set at 120 mL/min and the temperature was increased from 30 to 800 °C at a rate of 10 °C/min. X-Ray photoelectron spectroscopy (XPS) was collected on an ESCALab MKII X-ray photoelectron spectrometer, using non-monochromatized Al_Kα_ X-ray as excitation source. The binding energies were corrected for specimen charging by calibrating the C_1s_ peak to 284.6 eV. Element analysis (EA) was characterized by element analyzer (German Heraeus Company, CHN-0-Rapid). The percentage composition of cobalt was analyzed by Inductively Coupled Plasma (America PE Company, Optima 5300 DV).

### Electrochemical measurements

The electrochemical measurements were performed in a conventional three electrode cell using a CHI760D (Shanghai Chenhua, China) electrochemical workstation with catalyst-coated glassy carbon (GC) as the working electrode, a Pt wire as the counter electrode and a saturated Ag/AgCl electrode as the reference electrode. The catalyst ink was prepared by blending 5 mg of the catalyst with 50 μL of Nafion solution (4 wt %) and 950 μL of water/isopropanol solution (3:1) via sonication. An amount of 5 μL of the dispersion was transferred onto the GC electrode with a catalyst loading of about 0.18 mg/cm^2^. Then, the prepared catalyst electrode was dried at room temperature. With this electrode as the working electrode, electrochemical measurements were conducted in 1 M KOH solution. Polarization curves were obtained at a scan rate of 1 mV/s. Accelerated degradation measurement was conducted for 3000 cyclic voltammetry (CV) cycles at a scan rate of 50 mV/s. In all measurements, the reference electrode was calibrated with respect to reversible hydrogen electrode (RHE). All Polarization data were without iR-corrected.

## Results and Discussion

**Figure 1:**
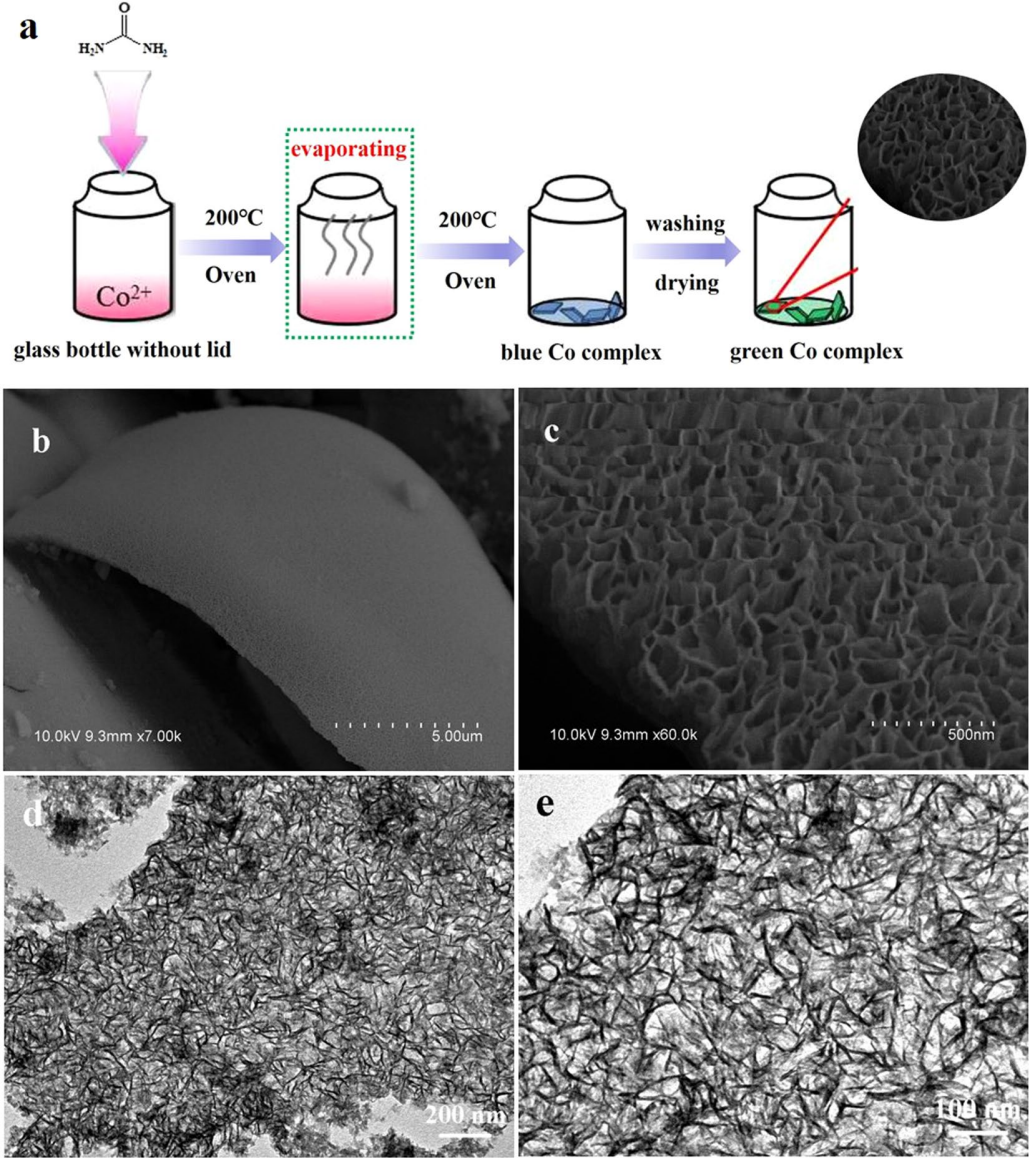
(**a**) The facile synthesis process of the 3D Co complex architecture; (**b**,**c**) SEM and (**d**,**e**) TEM images of the as-synthesized product.

**Figure 2:**
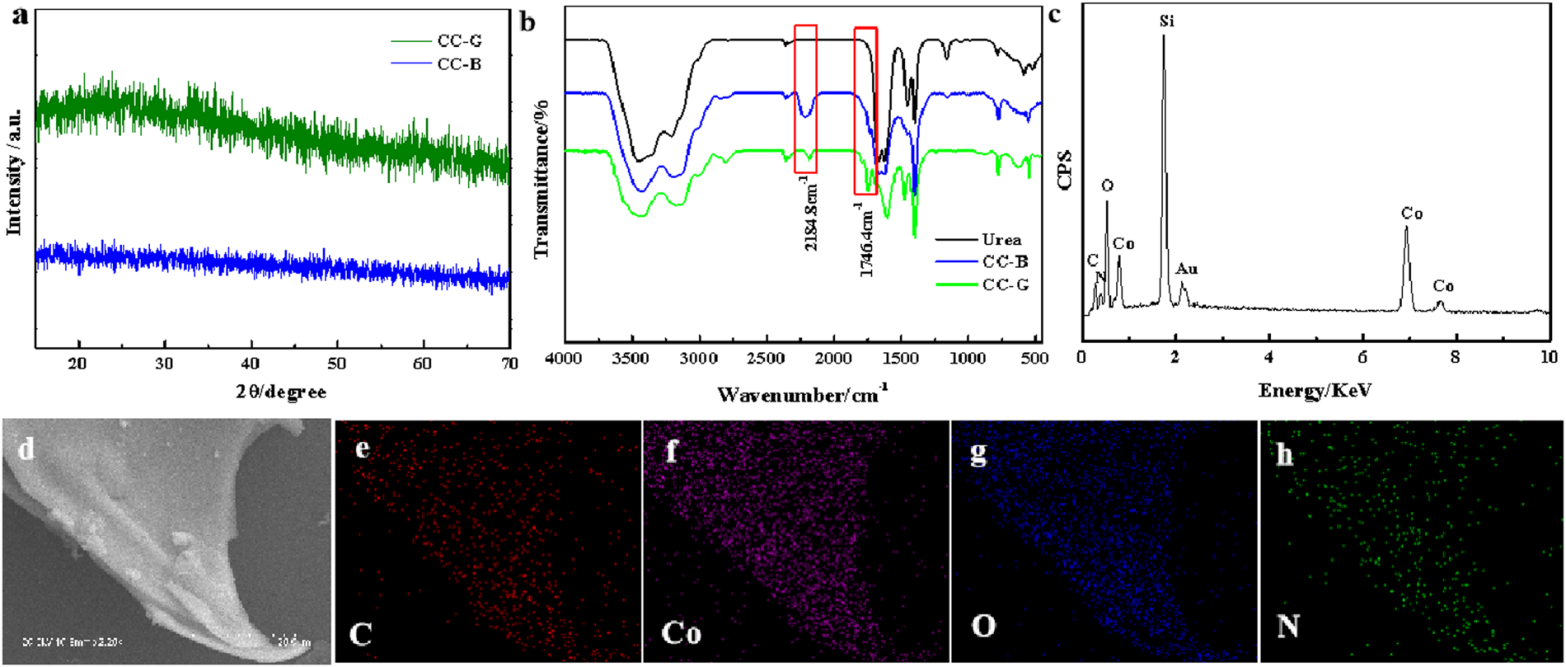
(**a**) XRD patterns; (**b**) IR spectra; (**c**) EDS pattern and (**d**~**h**) elemental mappings of the as-synthesized product.

Figures 1a and S1 briefly depict the facile synthesis process of the 3D Co complex. Figure 1b,c show SEM images of the product, revealing that the sample consists of plate-like 3D superstructures. The 3D plate is assembled by a great number of 2D nanosheets, which are curled and perpendicular to the plate surface, resulting in a lot of porous channels. TEM images in Fig. 1d,e also confirm the 3D porous superstructure of the product constructed by thin 2D nanosheets. Figure S2 shows the adsorption/desorption isothermals and the pore size distribution of the obtained Co-complex architecture, which clearly confirm the mesoporous structure with the pore sizes in the range of 3~12 nm. Since the solvent evaporation process is very quick and there is no enough time for slow crystallization in solution, no obvious diffraction peaks can be detected in the XRD patterns (Fig. 2a), suggesting the amorphous nature of the obtained Co product. Fourier transform infrared spectra (FTIR) is employed to analyze the information of functional groups. Figure 2b shows the FTIR spectra of the synthesized products and pure urea. It is obviously observed that both CC-B and CC-G have similar curves to urea, suggesting the remaining existence of urea molecules in the product, which would serve as ligands to coordinate with Co^2+^. Newly emerged peak at 2184.8 cm^−1^ indicates the coordination bonding between Co^2+^ and ligands (urea). Compared to that of urea, stretching vibration of C-N in the product nearly disappears, suggesting possible interaction between Co^2+^ and amino group that weakens the vibration of C-N. The specific peak at 1746.4 cm^−1^ in CC-G may result from coordination bond of Co-O by the hydrolysis of CC-B and water. Energy dispersive spectrum (EDS) in Fig. 2c further proves the co-existence of multiple elements including C, O, N and Co (Si from substrate and Au from gold sputtering), confirming the obtained product could be a Co-based complex with the organic ligand. Elemental mappings (Fig. 2d~h) verify that the product is made up of C, O, N and Co in accordance with the EDS analysis, and these elements are uniformly dispersed in the product, suggesting that the product obtained through the fast solvent evaporation process is not cobalt oxides or hydroxides. It can be concluded by combining the XRD, IR and EDS results that the as-prepared product would be amorphous Co complex through the coordination between Co^2+^ and urea.

**Figure 3:**
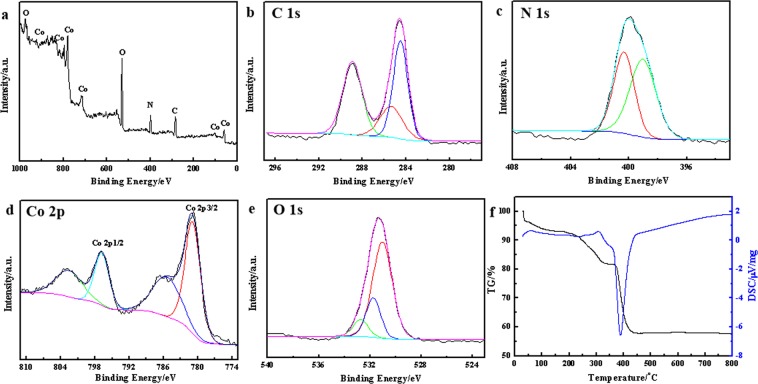
(**a**) Survey XPS spectrum; (**b**~**e**) High resolution XPS spectra of (**b**) C1s, (**c**) N1s, (**d**) Co2p, (**e**) O1s and (**f**) TG-DTA and DSC curves of the as-synthesized product.

Since the OER mainly occurs on the surface of catalyst, it is necessary to study the surface properties with X-ray photoelectron spectroscopy (XPS). All of the binding energies in XPS were corrected for specimen charging by referencing to the C1s peak (set at 284.6 eV). Co, C, N and O were all detected from the overall XPS spectrum of the 3D Co complex in Fig. 3a. Figure 3b shows the fine-scanned C1s spectrum, in which the peaks of C1s locates at around 288.9 eV and 285.4 eV contributing to the carbonyl and C-N. Figure 3c shows that the sample has a obvious peak located at 399.9 eV, which should be attributed to the Co-N according to the NIST XPS data. The Co2p XPS spectrum is shown in Fig. 3d, in which the peaks of Co 2p^3/2^ and Co2p^1/2^ are at around 781.0 eV and 796.9 eV, respectively, with a ∆E_3/2-1/2_ of 15.9 eV, indicating that the state of cobalt could be Co^2+^. The O1s XPS spectrum in Fig. 3e shows three peaks locating at 532.7 eV, 531.7 eV, and 531 eV, which indicate the existence carbonyl oxygen and hydroxy oxygen. Inductively coupled plasma spectroscopy (ICP) and elemental analyzer (EA) are regarded as efficient means to characterize the content of elements. The ICP measurement demonstrates that the mass fraction of Co in Co complex is about 42.75%, and EA measurement shows the approximate atomic ratio of C:N:H is 1:1:3. Combining the existing urea, hydroxy, and possible carbonate according to the XPS and FTIR data, the structural formula of the Co-based complex can be inferred as Co_2_(CN_2_H_4_O)(OH)_2_CO_3_, which has a Co content of 43.06% in accordance with the ICP and EA results. Figure 3f presents the thermogravimetric analysis (TGA) and differential scanning calorimeter (DSC) curves of the Co complex under air atmosphere. After the decomposition and the oxidation reaction of Co^2+^ to Co^3+^, the sample has a remaining mass of 57.8% of initial mass, which is in accordance with the result of ICP and demonstrates the rationality of the proposed structural formula of the Co-based complex.

The formation of 3D Co_2_(CN_2_H_4_O)(OH)_2_CO_3_ architecture goes through the following two steps: Firstly, the free Co ions react with urea and CO_3_^2−^ produced by the decomposition of urea to form blue bulk Co complex with constant evaporation of solvent at 200 °C under open system, and secondly, the blue bulk Co complex can be transferred to green 3D Co complex architecture assembled by nanosheets through hydrolysis process by washing the blue Co complex with deionized water. The first step is the key for the formation of the 3D Co_2_(CN_2_H_4_O)(OH)_2_CO_3_ structure. The rapid solvent evaporation under high temperature prevents the total decomposition of the used urea. The remained urea can serve as ligand to coordinate with Co^2+^ in the following procedures. At the same time, the decomposition of part of urea results in the formation of CO_3_^2−^ to balance the positive charges. The Co complex tends to form dense bulk in the concentrated system, then the 2D nanosheets produced by hydrolysis directly grow on the blue bulk for final 2D nanosheets-assembled 3D architecture. So, it is highly necessary to set high temperature condition for the rapid evaporation of solvent for the formation of 3D architecture. Conversely, relatively slow solvent-evaporation experiments at 100 °C and 150 °C were also conducted. Figure S3 shows the SEM images of the obtained products at 100 °C (SE-100) and 150 °C (SE-150), which both present disordered distribution of bulk nanoplates. XRD patterns (Fig. S4a) confirm that they are also amorphous in nature and the corresponding XPS spectra (Fig. S4b) demonstrate that the state of cobalt are also Co^2+^. However, the most of urea, which serve as ligand to form complex in RSE system, gradually decomposes into NH_3_ and CO_2_ in the relatively long time under low temperatures. Therefore, the 2D nanosheets-assembled 3D Co_2_(CN_2_H_4_O)(OH)_2_CO_3_ complex cann't be realized under low temperatures because of the absence of ligands.

**Figure 4:**
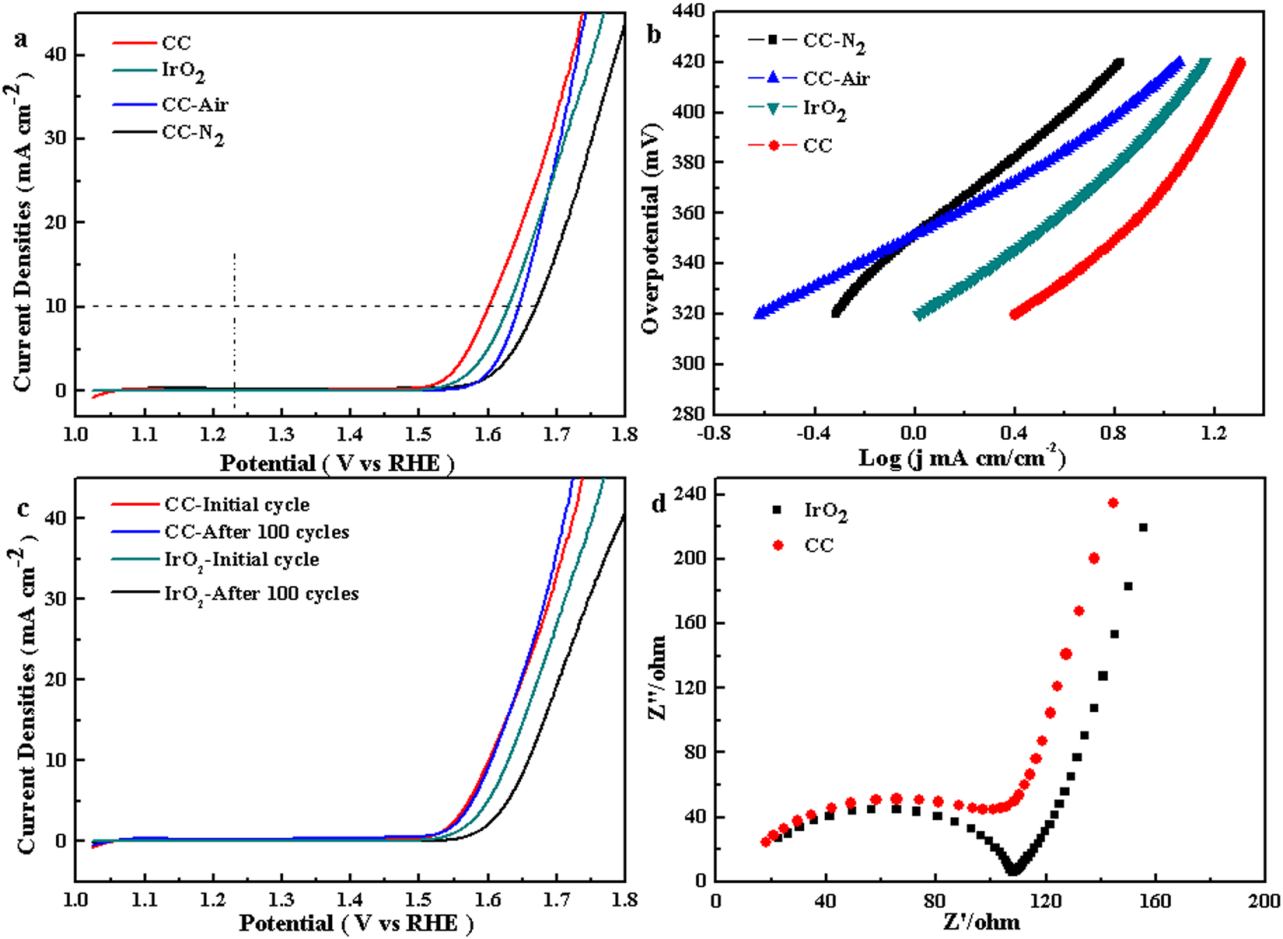
(**a**) Polarization curves of different Co-based products and commercial IrO_2_ in 0.1 M KOH aqueous solution; (**b**) The corresponding Tafel plots derived from polarization curves; (**c**) Polarization curves of the 3D Co complex architecture and commercial IrO_2_ before and after cyclic voltammogram test for 100 cycles in 0.1 M KOH solution; (**d**) AC impedance spectra of the 3D Co complex architecture and commercial IrO_2_.

**Figure 5:**
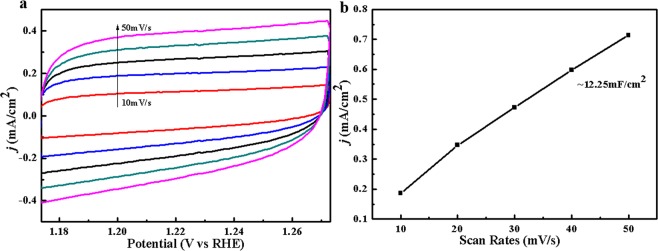
(**a**) CVs of CC in 1 M KOH at different scan rates from 10 to 50 mV/s; (**b**) Charging current density differences (Δj = j_a_ − j_c_) at 1.20 V vs. RHE plotted against the scan rate. The linear slope (equivalent to twice of the C_dl_) was used to represent the ECSA.

The OER electrocatalytic activity were investigated in 1 M KOH aqueous solution using a standard three-electrode system. As shown in Fig. 4a, initial linear sweep voltammetry (LSV) curves recorded with the Co complex (CC) reveals a relatively lower overpotential of 360 mV at a current density of 10 mA/cm^2^ than that of commercial IrO_2_ (400 mV) under the same conditions. Meanwhile, to highlight the superior OER performance, Co_3_O_4_ (CC-air) and Co_3_C_x_ (CC-N_2_) nanostructures were also synthesized by calcining the 3D Co complex under air or N_2_ atmosphere. XRD patterns, IR, XPS spectra and SEM images shown in Figs S5 and S6 clearly demonstrate the complete transformation from Co complex to Co_3_O_4_ and Co_3_C_x_. The polarization curves of Co_3_O_4_ (CC-air) and Co_3_C_x_ (CC-N_2_) nanostructures are also displayed in Fig. 4a, whose onset potentials and overpotentials are higher than those of Co complex or IrO_2_. The Tafel slope derived from polarization (Fig. 4b) was used to evaluate OER kinetics. Co complex has an approximately equivalent Tafel slope to commercial IrO_2_, indicating the excellent OER activity at low overpotential. It is worth mentioning that the obtained Co_3_O_4_ has a lowest Tafel slope, contributing to an even higher enhancement in OER activity at high potential. Worse OER performance of magnetic CoC_x_ may be caused by the deletion of oxygen-containing functional groups under high temperature condition, which benefits the absorption of H_2_O molecules on the catalyst. LSV curves after 100 CV cyclings at a scan rate of 0.1 V·s^−1^ were utilized to compare the stability of 3D Co complex and IrO_2._ Figure 4c shows the almost completely coincident LSV curves, indicating the excellent stability for Co complex. The corresponding SEM images, XRD pattern and IR spectrum of the catalyst after 100 OER cycles shown in Fig. S8 demonstrate that the microstructure and composition of Co complex are highly stable. In contrast, the LSV of IrO_2_ after 100 CV cyclings has a big deviation. Figure S9a shows the LSV curves of 3D Co complex, IrO_2_ and amorphous SE-100 after 1000 CV cyclings, which indicates the 3D Co complex has a remarkably superior performance for OER than IrO_2_ or SE-100 in the long-term cycle testing, though it has a fine deviation compared to initial cycle that might result from possible fall off of catalyst on the surface of electrode. AC impedance spectra for Co complex and IrO_2_ displayed in Fig. 4d indicate the low electron transfer impedance (R_ct_) for Co complex_._ Impedance data demonstrate that charge transfer and ions diffusion are not advantages versus that of IrO_2_. It is well accepted that the electrochemically active surface area (ECSA) of the catalysts can be estimated from measurements of the electrochemical double-layer capacitance (C_dl_)^12,14^. The double layer capacitance (C_dl_), which can calculated by using cyclic voltammetric method, is proportional to ECSA. The bigger the effective electrochemical surface area, the more of the active sites. Figure 5a shows the CVs of the CC products, which reveals that the sample has an obvious increase in current density according to different scan rates. The calculated EDLC of CC is 12.25 mF/cm^2^ (Fig. 5b), superior to the reported OER electrocatalysts^12,14^. The result indicates that the number of electrochemically active sites for water oxidation significantly increases due to the special structure. Compared with the control samples, CC has a highest specific surface area of 142.9 m^2^/g (Table S1). It is believed that the initial OER process in alkaline conditions involves adsorbed OH and O species on the active sites of catalysts based on the following scheme^32^.

1 $${{\rm{O}}{\rm{H}}}^{-}+\ast \to {{\rm{O}}{\rm{H}}}^{\ast }+{{\rm{e}}}^{-}$$

2 $${{\rm{O}}{\rm{H}}}^{\ast }+{{\rm{O}}{\rm{H}}}^{-}\to {{\rm{O}}}^{\ast }+{{\rm{H}}}_{2}{\rm{O}}+{{\rm{e}}}^{-}$$

3 $${{\rm{O}}}^{\ast }+{{\rm{O}}{\rm{H}}}^{-}\to {{\rm{O}}{\rm{O}}{\rm{H}}}^{\ast }+{{\rm{e}}}^{-}$$

4 $${{\rm{O}}{\rm{O}}{\rm{H}}}^{\ast }+{{\rm{O}}{\rm{H}}}^{-}\to {{\rm{O}}}_{2}+{{\rm{H}}}_{2}{\rm{O}}+{{\rm{e}}}^{-}$$

$${\rm{S}}{\rm{u}}{\rm{m}}{\rm{m}}{\rm{a}}{\rm{r}}{\rm{y}}:4{{\rm{O}}{\rm{H}}}^{-}\to {{\rm{O}}}_{2}+2{{\rm{H}}}_{2}{\rm{O}}+4{{\rm{e}}}^{-}$$

Step (1) and (2) play an important role to determine the overall OER rate, while reactions (3), (4) for O_2_ production are inversible and fast^33^. Therefore, the adsorption energy of OH^−^ greatly affects the OER process. 2D nanosheets arrays with abundant hydrophilic functional groups have high specific surface area for the contact of catalyst and OH^−^ or H_2_O. Meanwhile, amorphous structure supplied abundant defect sites for OER^34–36^. More importantly, the self-assembly of 2D nanosheets for stable 3D architecture that avoids the undesirable re-stack and condense of 2D nanosheets can remarkably improve the duration performance of catalyst for OER.

## Conclusions

In summary, we firstly proposed an extremely facile route called “rapid solvent-evaporation strategy” to prepare 3D Co complex architectures constructed by 2D nanosheet blocks as highly efficient catalyst of OER. The new-type Co-based catalyst exhibited excellent electrocatalytic activity of OER with a low onset potential and a lower overpotential of 360 mV at a current density of 10 mA·cm^−2^ than that of commercial IrO_2_. Abundant active sites and hydrophilic group of 2D nanosheets, amorphous and stable 3D structure, cooperatively achieved the superior catalyzing OER performance. It is expected that the 3D Co complex prepared by simple, green and low-cost method would be as promising OER catalyst for large-scale application to replace expensive and earth-rare IrO_2_. Meanwhile, it is believed that through the simple “rapid solvent-evaporation” strategy other novel 3D structures with great application potential could be obtained in the future.

## Supplementary information

Supporting information

## References

[1] Zare M (2018). Evolution of rough-surface geometry and crystalline structures of aligned TiO2 nanotubes for photoelectrochemical water splitting. Sci. Rep..

[2] Naseri N (2017). Microstructure, morphology and electrochemical properties of Co nanoflake water oxidation electrocatalyst at micro- and nanoscale. RSC Adv..

[3] Naseri N (2018). How morphological surface parameters are correlated with electrocatalytic performance of cobalt-based nanostructures. J. Ind. Eng. Chem..

[4] Wang JH (2016). Recent progress in cobalt‐based heterogeneous catalysts for electrochemical water splitting. Adv. Mater..

[5] Sun YF (2014). Atomically-thin non-layered cobalt oxide porous sheets for highly efficient oxygen-evolving electrocatalysts. Chem. Sci.

[6] Yuan CZ (2016). Cobalt phosphate nanoparticles decorated with nitrogen-doped carbon layers as highly active and stable electrocatalysts for the oxygen evolution reaction. J. Mater. Chem. A.

[7] Kanan MW, Nocera DG (2008). In situ formation of an oxygen-evolving catalyst in neutral water containing phosphate and Co^2+^. Science.

[8] Xu L (2016). Plasma-engraved Co_3_O_4_ nanosheets with oxygen vacancies and high surface area for the oxygen evolution reaction. Angew. Chem. Int. Ed..

[9] Rosen J, Hutchings GS, Jiao F (2013). Ordered mesoporous cobalt oxide as highly efficient oxygen evolution catalyst. J. Am. Chem. Soc..

[10] Zou XX, Goswami A, Asefa T (2013). Efficient noble metal-free (electro)catalysis of water and alcohol oxidations by zinc-cobalt layered double hydroxide. J. Am. Chem. Soc..

[11] Zhao ZL, Wu HX, He HL, Xu XL, Jin YD (2014). A high‐performance binary Ni-Co hydroxide-based water oxidation electrode with three-dimensional coaxial nanotube array structure. Adv. Funct. Mater..

[12] Jiang YM, Li X, Wang TX, Wang CM (2016). Enhanced electrocatalytic oxygen evolution of α-Co(OH)_2_ nanosheets on carbon nanotube/polyimide films. Nanoscale.

[13] Popczun EJ, Read CG, Roske CW, Lewis NS, Schaak RE (2014). Highly active electrocatalysis of the hydrogen evolution reaction by cobalt phosphide nanoparticles. Angew. Chem. Int. Ed..

[14] Liu YY (2015). Electrochemical tuning of olivine-type lithium transition-metal phosphates as efficient water oxidation catalysts. Energy Environ. Sci..

[15] Sun YJ (2013). Electrodeposited cobalt-sulfide catalyst for electrochemical and photoelectrochemical hydrogen generation from water. J. Am. Chem. Soc..

[16] Liu YW (2014). Low overpotential in vacancy-rich ultrathin CoSe_2_ nanosheets for water oxidation. J. Am. Chem. Soc..

[17] Huang JH (2015). CoOOH nanosheets with high mass activity for water oxidation. Angew. Chem. Int. Ed..

[18] Zhang B (2016). Homogeneously dispersed multimetal oxygen-evolving catalysts. Science.

[19] Jiang M, Li YJ, Lu ZY, Sun XM, Duan X (2016). Binary nickel-iron nitride nanoarrays as bifunctional electrocatalysts for overall water splitting. Inorg. Chem. Front..

[20] Zhong X (2015). Synergistic effect of nitrogen in cobalt nitride and nitrogen-doped hollow carbon spheres for the oxygen reduction reaction. ChemCatChem.

[21] Yoon KR (2018). Brush-like cobalt nitride anchored carbon nanofiber membrane: Current collector-catalyst integrated cathode for long cycle Li-O_2_ batteries. ACS Nano.

[22] Ullman AM, Brodsky CN, Li N, Zheng SL, Nocera DG (2016). Probing edge site reactivity of oxidic cobalt water oxidation catalysts. J. Am. Chem. Soc..

[23] Bergmann A, Martinez-Moreno E, Teschner D, Chernev P, Gliech M (2015). Reversible amorphization and the catalytically active state of crystalline Co_3_O_4_ during oxygen evolution*Nat*. Commun..

[24] Kataoka Y (2009). Photocatalytic hydrogen production from water using porous material [Ru_2_(p-BDC)_2_]_n_. Energy Environ. Sci..

[25] Levchenko TI (2015). Controlled solvothermal routes to hierarchical 3D superparticles of nanoscopic CdS. Chem. Mater..

[26] Ming T (2008). Ordered gold nanostructure assemblies formed by droplet evaporation. Angew. Chem. Int. Ed..

[27] Wang PP (2015). Zinc sulfide nanosheet‐based hybrid superlattices with tunable architectures showing enhanced photoelectrochemical properties. Small.

[28] Dang LY, Wei CZ, Ma HF, Lu QY, Gao F (2015). Three-dimensional honeycomb-like networks of birnessite manganese oxide assembled by ultrathin two-dimensional nanosheets with enhanced Li-ion battery performances. Nanoscale.

[29] Li HY, Wang X (2015). Three-dimensional architectures constructed using two-dimensional nanosheets. Sci. China Chem..

[30] Liao CW, Lin YS, Chanda K, Song YF, Huang MH (2013). Formation of diverse supercrystals from self-assembly of a variety of polyhedral gold nanocrystals. J. Am. Chem. Soc..

[31] Zhao Y (2009). Small-molecule-directed nanoparticle assembly towards stimuli-responsive nanocomposites. Nat. Mater..

[32] Huang W, Zuo ZJ, Han PD, Li ZH, Zhao TD (2009). XPS and XRD investigation of Co/Pd/TiO_2_ catalysts by different preparation methods. J. Electron. Spectrosc..

[33] Bajdich M, García-Mota M, Vojvodic A, Nørskov JK, Bell AT (2013). Theoretical investigation of the activity of cobalt oxides for the electrochemical oxidation of water. J. Am. Chem. Soc..

[34] Xu K (2015). Metallic nickel nitride nanosheets realizing enhanced electrochemical water oxidation. J. Am. Chem. Soc..

[35] Yang Y, Fei HL, Ruan GD, Xiang CS, Tour JM (2014). Efficient electrocatalytic oxygen evolution on amorphous nickel-cobalt binary oxide nanoporous layers. ACS Nano.

[36] Smith RDL (2013). Photochemical route for accessing amorphous metal oxide materials for water oxidation catalysis. Science.

