# Supplementary material for: Rapid solvent-evaporation strategy for three-dimensional cobalt-based complex hierarchical architectures as catalysts for water oxidation
Source: Sci Rep. 2019 Oct 30;9:15681. doi: 10.1038/s41598-019-51979-z (PMC6821758; doi:10.1038/s41598-019-51979-z)
Supplement: Supplementary file 1 — Supporting information [file 41598_2019_51979_MOESM1_ESM.doc]

**Supporting Information (SI)**

**Rapid solvent-evaporation strategy for three-dimensional cobalt-based complex hierarchical architectures as catalysts for water oxidation**

Hong Jiang, Hao Zhang, Qiaoling Kang, Haifeng Ma, Yinlin Tong, Feng Gao, Qing yi Lu


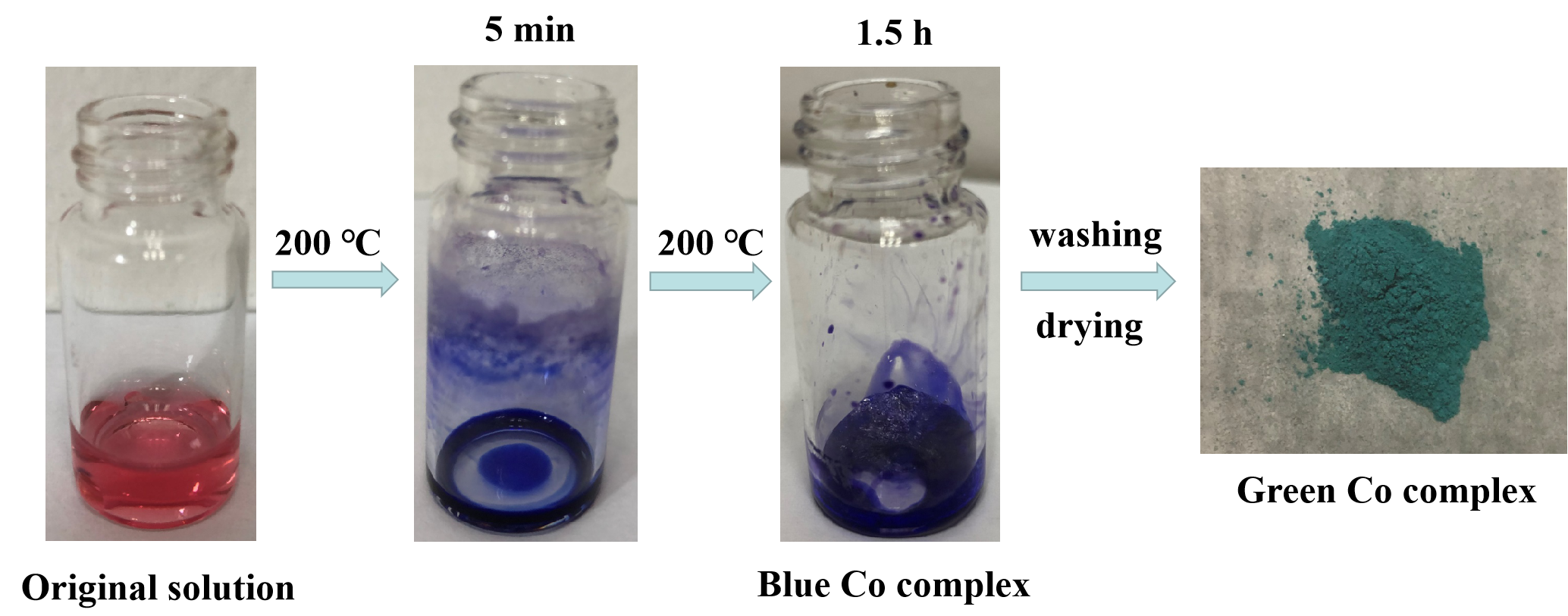


**Figure S1** Schematic diagram of experimental preparation process


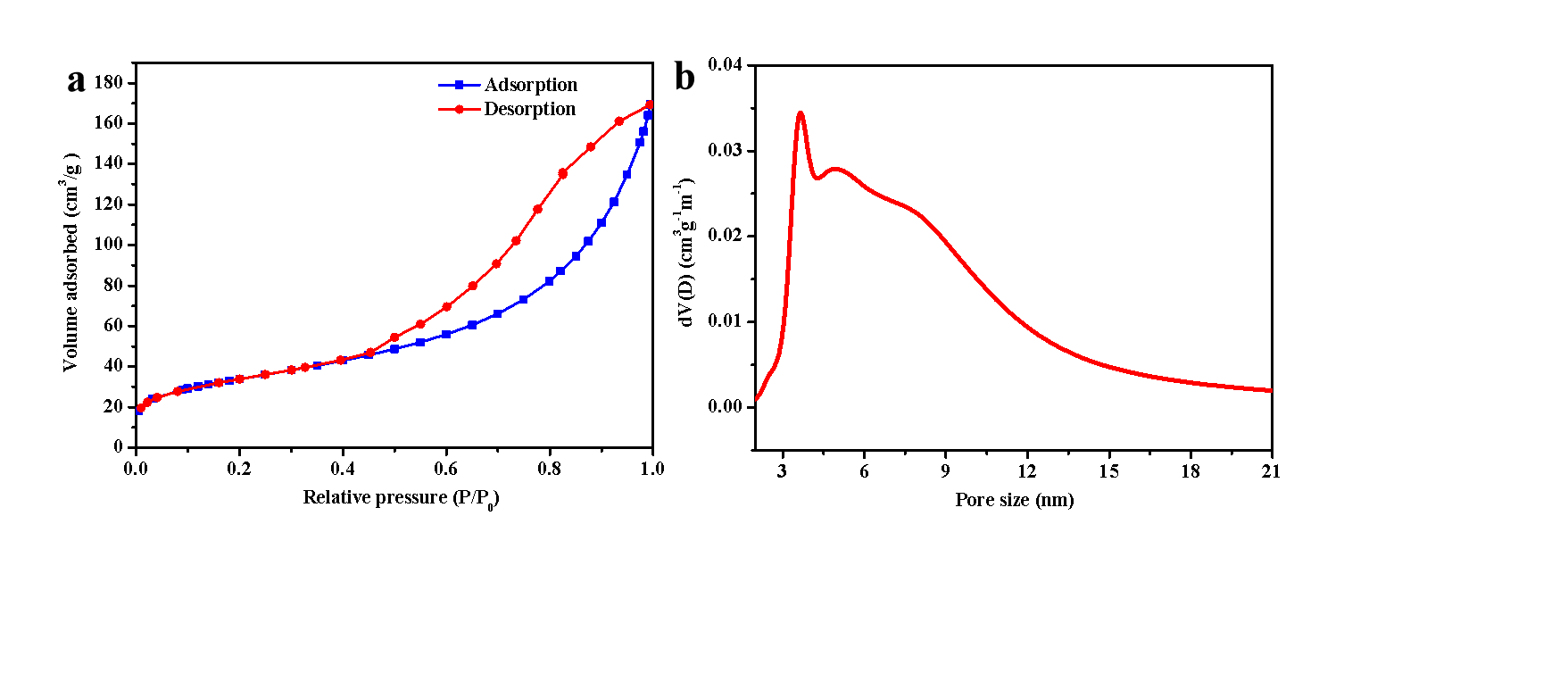


**Figure S2** (a) Nitrogen adsorption-desorption isotherms and (b) Pore size distribution of 3D Co complex architecture

**Figure S3** SEM images of the products prepared under slow-evaporation conditions: (a) 100 °C and (b) 150 °C.


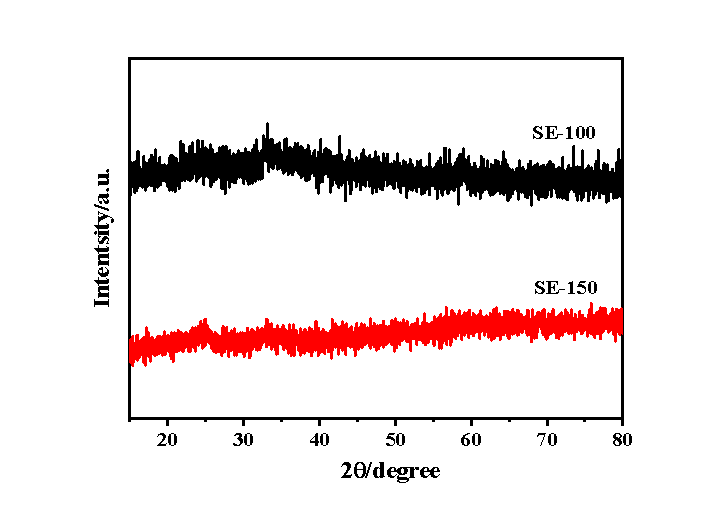


**Figure S4** XRD patterns of the products prepared under slow-evaporation conditions.


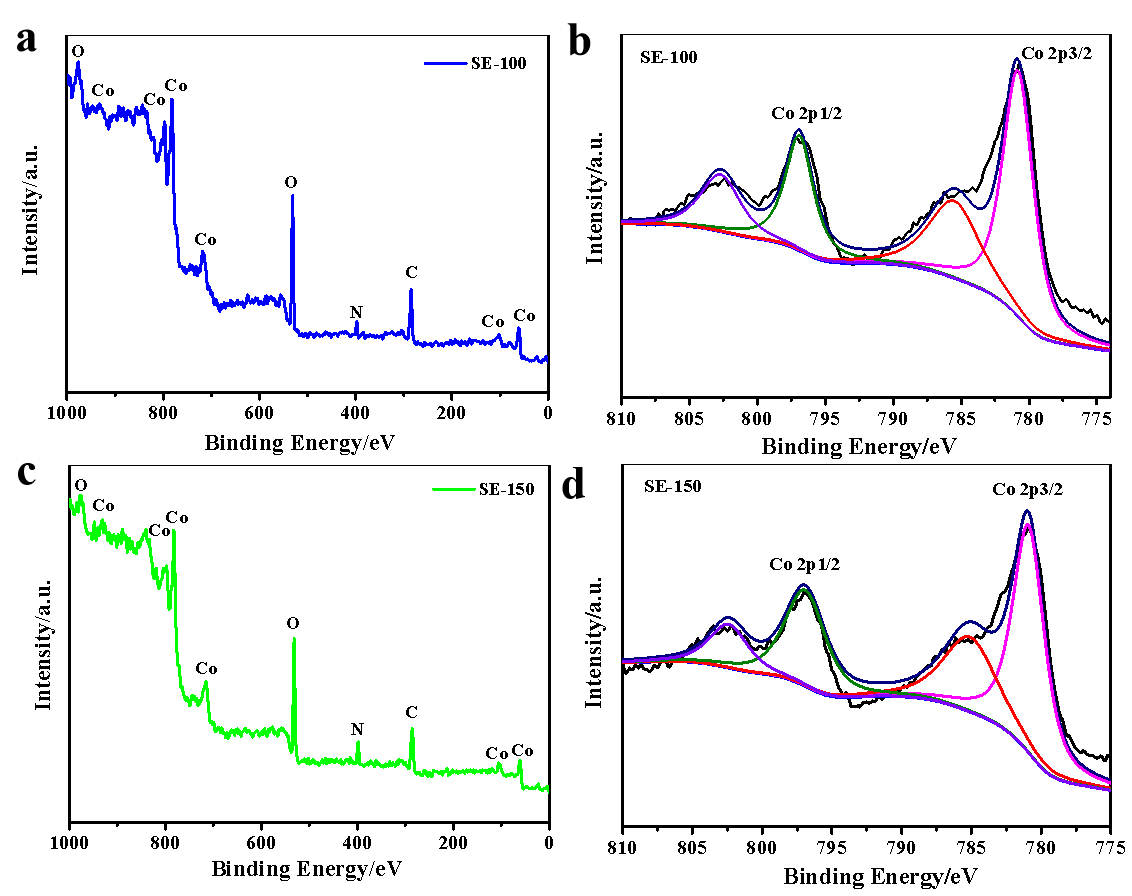


**Figure S5** Survey XPS and high resolution XPS spectra of Co of (a, b) SE-100 and (c, d) SE-150.


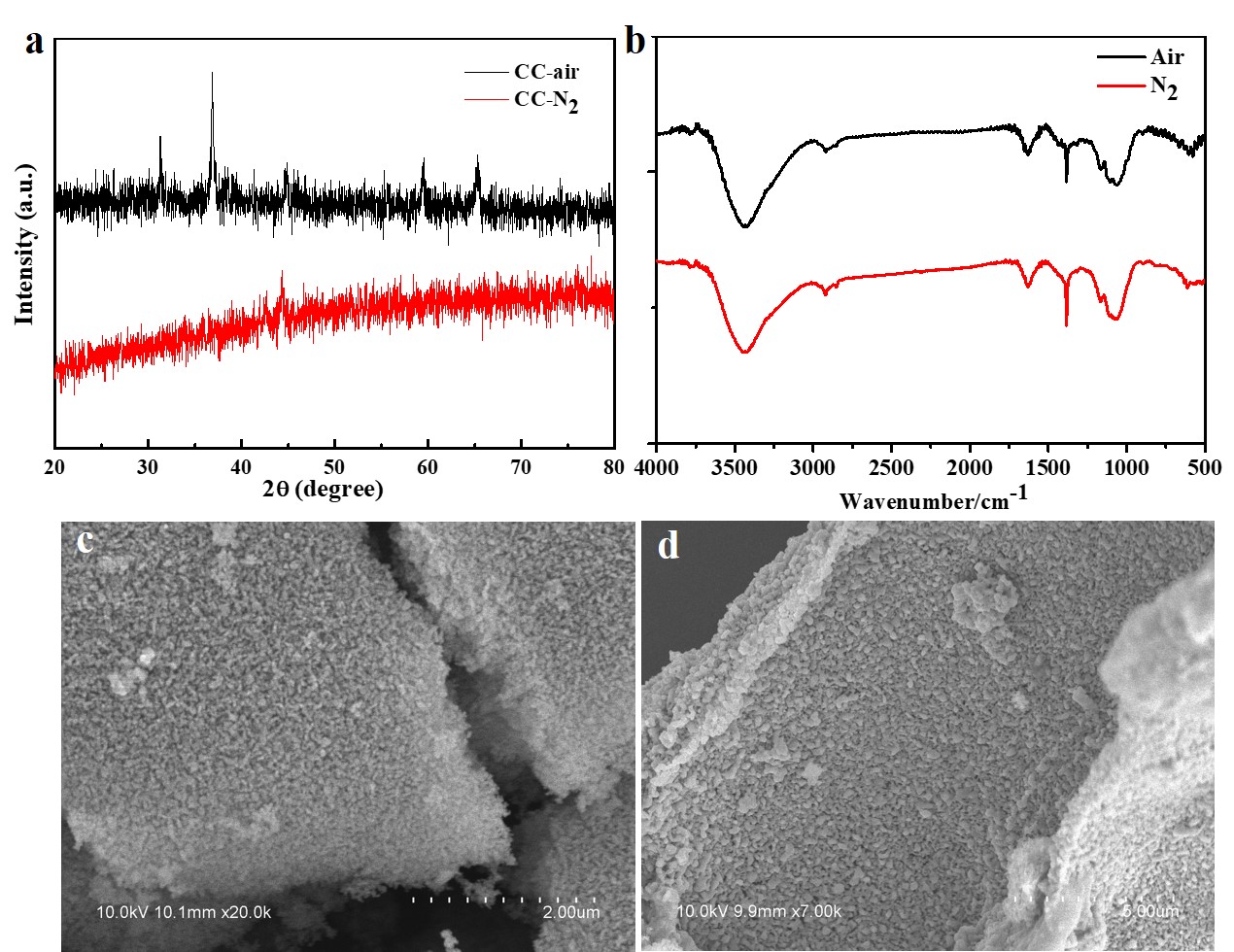


**Figure S6** (a) XRD patterns, (b) IR spectra of the products prepared by calcining the as-synthesized Co-based complex in air and N_2_ atmospheres; (c, d) SEM images of the products prepared by calcining the as-synthesized Co-based complex in (c) air and (d) N_2_ atmospheres.


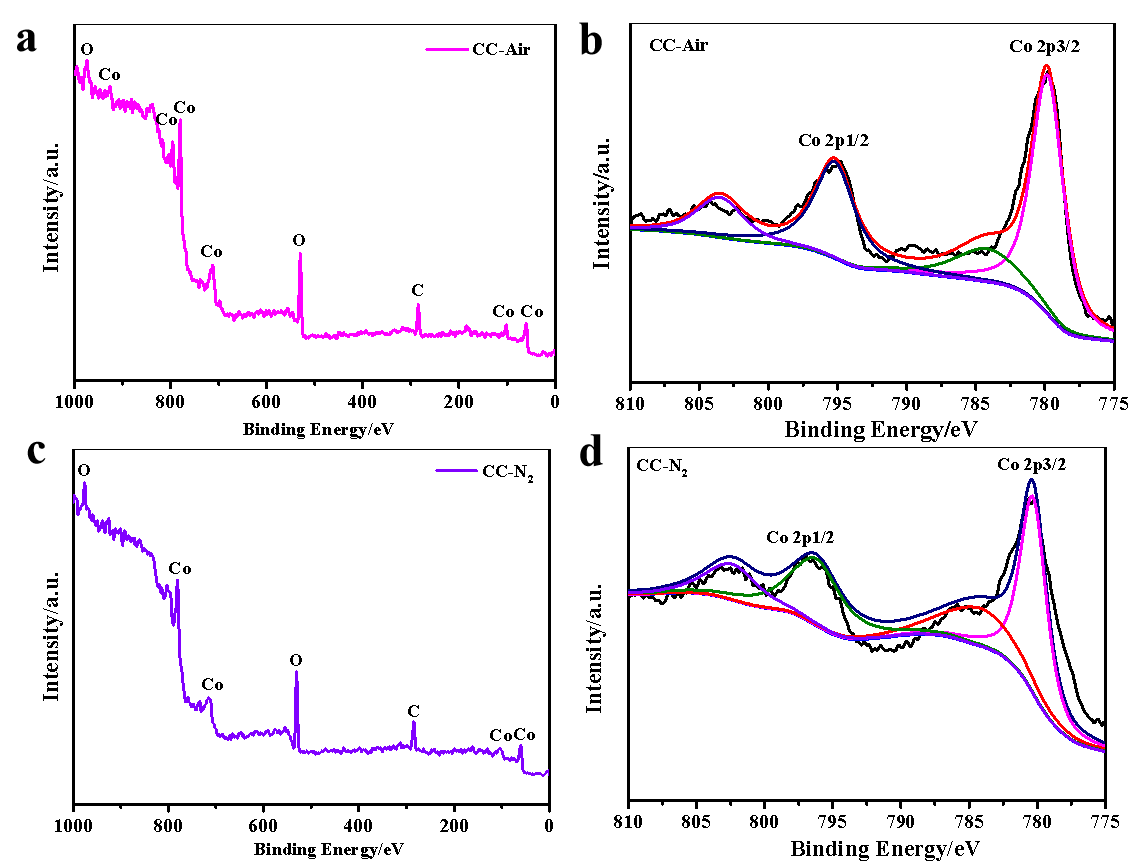


**Figure S7** Survey XPS and high resolution XPS spectra of Co of (a, b) CC-Air and (c, d) CC-N_2。_


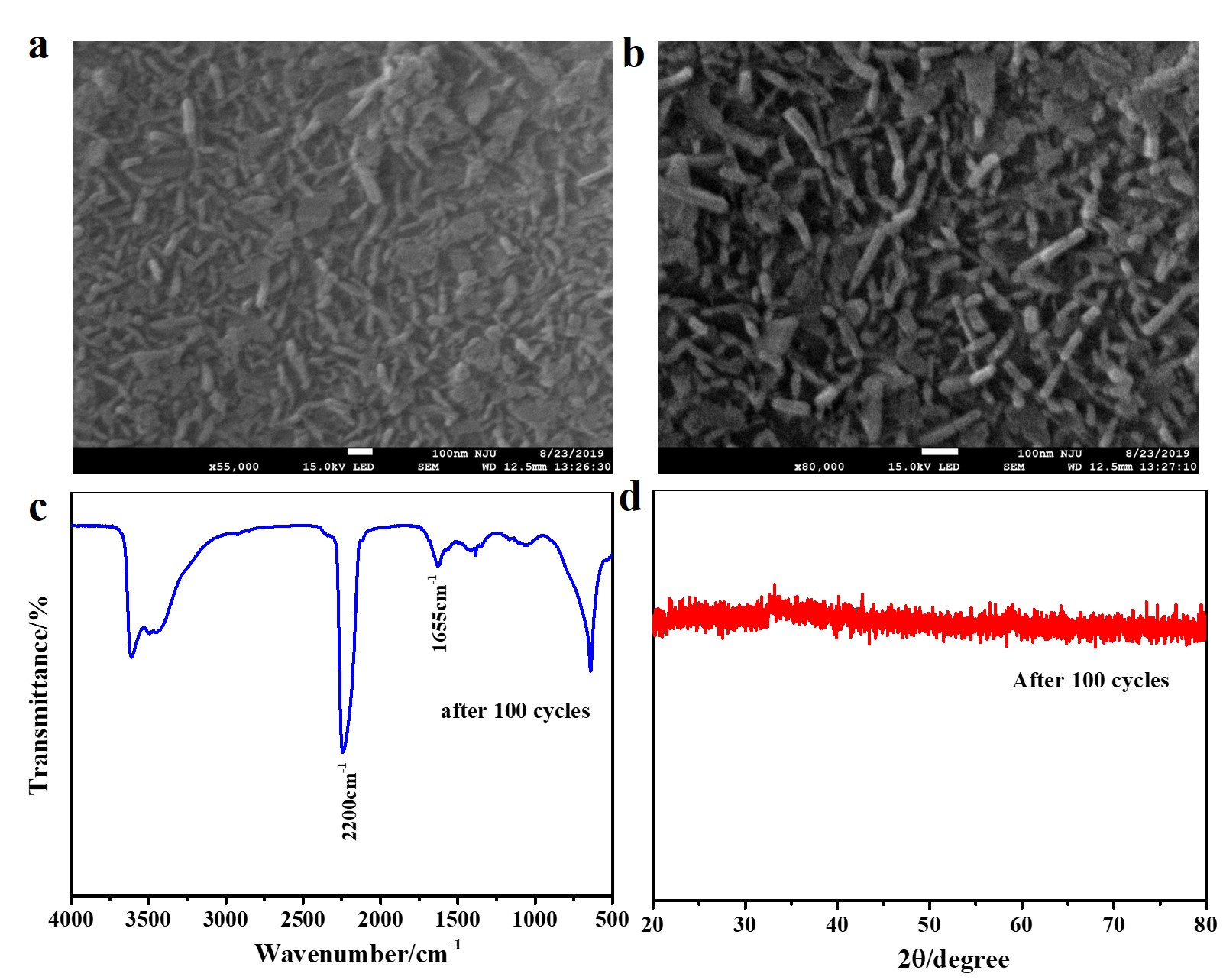


**Figure S8** (a, b) SEM images; (c) IR spectrum and (d) XRD pattern of the catalyst after 100 cycles of OER.

**Figure S9** (a) Polarization curves of the SE-100 prepared under slow evaporation conditions at 100 °C in 1 M KOH aqueous solution; (b) Polarization curves of the 3D Co complex architecture, SE-100 and commercial IrO_2_ after cyclic voltammogram test for 1000 cycles in 1 M KOH solution.

**Table S1** BET surface area, pore volume and pore size of the different samples

| **Samples** | **S_BET_ (m^2^ g^-1^)** | **Pore volume (cm^3^ g^-1^)** | **Pore size (nm)** |
| --- | --- | --- | --- |
| **SE-200 (CC-G)** | 142.9220 | 0.2708 | 7.58168 |
| **SE-100** | 51.8098 | 0.101988 | 7.87 |
| **SE-150** | 121.6080 | 0.23337 | 7.67 |
| **CC-Air** | 38.5109 | 0.091797 | 9.53 |
| **CC-N_2_** | 12.3302 | 0.019607 | 6.36 |

**Table S2** Comparison of the electrocatalytic activity of Co complex with recently-reported solid-state catalysts for OER in 1 M KOH electrolyte.

|  | Current density (j) | Overpotential (mV) | Reference |
| --- | --- | --- | --- |
| **Co complex** | **10 mA/cm^2^** | **370** | **This work** |
| Co_3_O_4_ | 10 mA/cm^2^ | 400 | *Nat. Chem.* **2011**, *3*, 79. |
| NiCo_2_Se_4_ | 10 mA/cm^2^ | 450 | *ACS Nano* **2017**, *11*, 9550-9557. |
| porous Co_3_O_4_ | 10 mA/cm^2^ | 420 | *Chem. Sci*. **2014**, *5*, 3976-3982 |
| Edge-rich, oxygen-functionalized graphene | 10 mA/cm^2^ | 450 | *Adv. Mater.* **2017**, *29*, 1606207*.* |
| CP/CNT/CoS | 10 mA/cm^2^ | 450 | *ACS Nano* **2016**, *10*, 2342-2348 |
| CoP hollow polyhedra | 10 mA/cm^2^ | 400 | *ACS Appl. Mater. Interfaces* **2016**, *8*, 2158-2165 |
| Fe_0.2_Ni_0.8_@N-GR | 10 mA/cm^2^ | 380 | *Adv. Funct. Mater.*  **2018**, *28*, 1706928 |
